# Investigation of the Surface Roughness and Surface Uniformity of a Hybrid Sandwich Structure after Machining

**DOI:** 10.3390/ma15207299

**Published:** 2022-10-19

**Authors:** Elżbieta Doluk, Anna Rudawska, Izabela Miturska-Barańska

**Affiliations:** Faculty of Mechanical Engineering, Lublin University of Technology, 20-388 Lublin, Poland

**Keywords:** circumferential milling, sandwich structure, aluminum alloy, CFRP composite

## Abstract

The parameters of surface roughness Ra, Rz and Rmax as well as surface topography Sa, Sz, Sp and Sv of the two-layer sandwich structure composed of an AW-2024 T3 aluminum alloy (Al) and a carbon-fiber-reinforced polymer (CFRP) were measured to determine an impact of the machining configuration (arrangement of the materials forming a sandwich structure) and the type of tool (presence of the tool coating) on the quality of the surface obtained through circumferential milling. The measurements revealed that milling produced different values of surface roughness for the aluminum alloy and the CFRP composite with values of 2D and 3D surface roughness being higher for the composite layer. The highest value of Ra of 1.10 µm was obtained for the surface of the CFRP composite using the CFRP/Al configuration and a TiAlN-coated tool. The highest values of the Rz (6.51 µm) and Rmax (8.85 µm) surface roughness parameters were also obtained for the composite layer using the same machining configuration and type of tool.

## 1. Introduction

A sandwich structure is made up of two basic components: a thinner external layer (facesheet), made of a stiffer material with better resistance properties, and a thicker but light internal layer (core), made of a different material. When joined together, the layers form a light, stiff, and durable structure which is stronger than similar monolithic structures [1,2,3]. Along with laminates, sandwich structures are classified as layered composites. Depending on their intended use, they can be made of different materials and can have different geometries and methods of stiffening and layer bonding. Sandwich structures can be divided into fully metal, hybrid, and composite structures, depending on the type of materials that make them up. Cavity milling is more difficult with hybrid layered composites than in the case of metal structures due to the heterogeneity and anisotropy of the structures resulting from materials with different mechanical and physical properties being bound together. In addition, bonding such materials requires considerable effort [4]. Moreover, sandwich structures are believed to be difficult to machine. During the machining of a sandwich structure composed of aluminum alloy and a carbon-fiber reinforced polymer (CFRP), it is additionally necessary to overcome difficulties related to the deformation of the upper and lower layers of the material, the interference of metal chips with the composite and the possible accumulation of aluminum shavings or carbon dust between the facings of the sandwich structure if it delaminates [5]. The heavy-duty carbon fibers used in composites are difficult to break and tend to drag, which very often causes them to develop micro-cracks and to delaminate along the operating direction of the tool [6]. Milling of CFRP composite requires frequent replacement of the milling tool, as the abrasive properties of carbon fiber cause premature wear. Furthermore, when machining CFRP composites, the milling tool alternately cuts hard fibers and the softer material of the skin, also accelerating the wear of the milling cutter [7,8].

Although hybrid layered structures are frequently used in many industries, the optimal conditions for machining this type of materials have not yet been adequately determined. Most studies focus on the damage to sandwich structures and their properties [2], compare their properties with those of similar monolithic structures [9], or review current trends in the use of such materials [10,11]. Additionally, recommendations on the machining of hybrid layered materials are based primarily on studies of CFRP composite processing.

Surface roughness is one of the most important and most commonly used properties that characterize the quality of a milled surface. It affects dimensional accuracy, alignment and the correct operation of machine and device parts [3,12,13]. Layered materials have been widely used in aerospace as structural components and parts of aircraft equipment. Components used in the aerospace industry must meet strict quality criteria due to its narrow tolerance ranges. Protective and decorative coatings are often applied to aviation components. High values of surface roughness parameters may result in insufficient adhesion of the coating and thus reduce its durability [14]. The heterogeneity and anisotropy of the machined material are also extremely important properties. The different surface roughness on the sandwich structure layers can significantly reduce the visual effect of the components. Therefore, it is important to obtain a uniform surface of the sandwich structure with the desired roughness and surface topography. Surface roughness is affected by multiple parameters, such as the level of wear of the cutting tool, the levels of vibration, the stability of the machining process, and the cutting parameters [15,16,17].

Many researchers have analyzed the roughness and geometric structure of a fiber composite surface after machining. However, no studies have been carried out to examine the roughness of milled hybrid sandwich structures. Some have noted [18,19] that milling strength and surface roughness during the machining of CFRP composites depend on the milling parameters, with feed rate being the main factor that affects the quality of the surface after machining. Suresh et al. [20] made similar conclusions. Ramulu et al. [21] conducted research on the machinability of CFRP composites. They noted that a higher cutting speed results in less surface roughness, which is supported by results published by Çolak et al. [22]. Channdrasekaran and Devarasiddappa [23] noted that the surface roughness of machined fiber composites is linearly dependent on the feed rate and inversely dependent on the cutting speed. Bayraktar et al. [24] determined the impact of the cutting tool (material, geometry, and tool coating) and the process parameters (cutting speed and feed rate) on the cutting speed and surface roughness of CFRP composites after milling. It was noted that feed rate had the greatest impact on the quality of the machined surface among the examined factors. A non-coated cutter produced the lowest cutting strength and surface roughness values, which increased with the number of flutes and the angle of inclination of the cutter’s cutting edge. Ramirez et al. [25] examined cutter wear and surface roughness after drilling CFRP composite. They concluded that new criteria must be formulated to assess surface topography after machining due to the anisotropy and heterogeneity of the machined material. Zarrouk et al. [26] examined the impact of machining conditions (rotational speed and machining depth) on the machining strength and surface quality of a milled nomex honeycomb. The results of the experiment were compared to a theoretical model. Eskandari et al. [27] studied the cutting speed, feed rate, and drill bit diameter in terms of their impact on the quality of openings drilled into sandwich structures with a foam core. Grilo et al. [28] investigated the delamination of drilled CFRP composites associated with drill bit diameter and machining parameters (feed rate and rotational speed). They showed that the feed rate was related directly by a proportional relationship to the delamination factor and the adjusted delamination factor. Shunmugesh et al. [29] used Grey Relational Analysis and the Taguchi technique to optimize machining conditions (machining speed, feed rate and drill bit diameter) in the context of delamination and surface quality (surface roughness and the dimensional accuracy of openings) after CFRP composite drilling. Khoran et al. [30] also studied the impact of machining parameters on the quality of openings produced by drilling. The experiment was performed on sandwich structures made of various materials (balsa wood, foamed materials) and used digital technology to measure the delamination and uncut fiber coefficients. Xu et al. [31] studied the effects of cutting parameters and tool geometry on surface roughness and cutting forces after milling of CFRP composites with a diamond tool (PCD). They showed that a higher value of tool rake angel did not affect surface roughness but resulted in lower cutting forces. Oláh et al. [32] reviewed solutions used in the cavity milling of sandwich structures and discussed the results of a simulation for cutting a honeycomb structure using a dedicated tool. The use of numerical methods such as the finite element method (FEM), the boundary element method (BEM), mathematical modeling, and the application of machine learning methods can help reduce the time and cost of testing, which is extremely important when testing relatively expensive composite materials [33,34]. Onyibo and Safaei [35] used the finite element method (FEM) to design sandwich structures with a honeycomb core. The study compared the mechanical properties obtained by modeling structures with different core thicknesses and made of different materials. Lavaggi et al. [36] studied the bonding of a honeycomb core with facesheets made of thermoset materials. They used three TGLM (Theory guided machine learning) models to optimize bonding properties such as the level of facesheets consolidation and bond-line porosity.

Milling an Al/CFRP structure causes heavy wear on the cutting tools, resulting in a poorer-quality machined surface. One way to obtain high-quality surfaces after milling is to use coated tools. Wang et al. [37] studied the tool wear during the drilling of CFRP/Ti structures. The wear of the cutter after machining a sandwich structure was compared against reference samples (used to machine separate composite and metal layers). The results indicated that the wear of the tool on the side of the metal layer was nine times lower than that on the side of the composite. The tests also found that each type of material resulted in a different type of wear. Hosokawa et al. [38] presented the results of CFRP composites milling using diamond-coated tools with a variable inclination angle of the cutting edge. They were able to demonstrate that the occurrence of defects on the surface of the machined material depended on machining forces, whereas tool wear depended on the orientation of the fibers.

The complexity of layered composites can cause many difficulties during the machining process. There are no comprehensive studies on the surface quality of sandwich structures after milling. There are also no standards or recommendations indicating an acceptable value or level of defect uniformity after milling of this type of constructions. Recommendations for cutting conditions (for example, recommended tool geometry, cutting parameters, machining configuration) have also not been developed. It causes machining conditions for this type of materials to be currently chosen based on recommendations for the composite layer, and cutting tools are dedicated for use with specific core types. This approach leads to a non-uniform surface quality of the sandwich structure, and this affects its further operation and aesthetic qualities. This study investigates the effect of the machining configuration (arrangement of layers forming the sandwich structure) and the type of tool (presence of a TiAlN coating) on the surface quality of the Al/CFRP sandwich structure after milling. The surface quality was defined by surface roughness and surface topography. A new and innovative approach is to evaluate the surface quality of the tested structure on the basis of the uniformity of defect distribution on the surface of individual layers of the structure. In addition, the I_R_ coefficient has been proposed as a tool for assessing the surface uniformity of such materials. The obtained research results are targeted for use in industrial practice. Prior to the study, it was assumed that circumferential milling of a two-layer metal–polymer composite sandwich structure results in differences in surface quality of the layers making up the sandwich structure. Based on the results obtained, the research hypothesis was verified, and the influence of the studied variables on the research object was determined.

## 2. Materials and Methods

Tests were performed on a two-layer sandwich structure made up of an EN AW-2024 T3 aluminum alloy (Al) and a carbon-fiber-reinforced polymer (CFRP) with an alternating arrangement of fibers. In the experiment, the cutting speed v_c_ [m/min], feed rate f_z_ [mm/blade], axial milling depth a_p_ [mm], radial milling depth a_e_ [mm], materials forming the structure, shape, and dimensions of the samples were used as constant factors. The output factors were surface roughness, surface topography, and surface uniformity. A two-level total plan was used to plan the experiment. The influence of two independent variables was studied: machining configuration and tool type. The design of the considered sandwich structure limited the machining configuration to the occurrence of two states. In order to limit the research time, two states were also adopted for the second independent variable (tool type). The applied method of planning the experiment made it possible to quickly generate all possible combinations of states of the independent variables.

The used aluminum alloy is characterized by low density and increased yield strength [39]. The alloy has low oxidation resistance, so it is not suitable for solutions where there is a risk of corrosion. The material is machinable but not suitable for anodizing and welding. One area of its application is the aerospace industry. The CFRP composite was created through a vacuum pressure impregnation process, using an autoclave manufactured by Scholz (Coesfeld, Germany). The proportion of carbon fibers in the hardened composite was around 60%. Table 1 shows selected properties of the CFRP composite used in the test.

Scotch-Weld EC-9323 B/A (3M, Saint Paul, MN, USA) epoxy glue was used to bond the aluminum alloy and the CFRP composite. The polymerization process took place in a vacuum bag under a pressure of 0.1 MPa for a period of 24 h. The plates were then seasoned under ambient conditions for 14 days.

**Figure 1:**
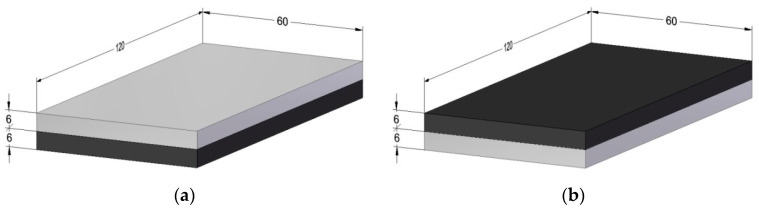
Tested sample: (**a**) Al/CFRP configuration, (**b**) CFRP/Al configuration (Unit: mm).

The machined samples had dimensions of 60 × 120 × 12 mm (Figure 1). The thickness of a single layer was 6 mm. Due to low value of the adhesive layer (0.1 ± 0.02 mm), it was disregarded when determining the total thickness of the structure. The experiment analyzed the impact of two machining configurations:

- Al/CFRP—milling starting from the metal layer (Figure 1a)
- CFRP/Al—milling starting from the composite layer (Figure 1b)

**Figure 2:**
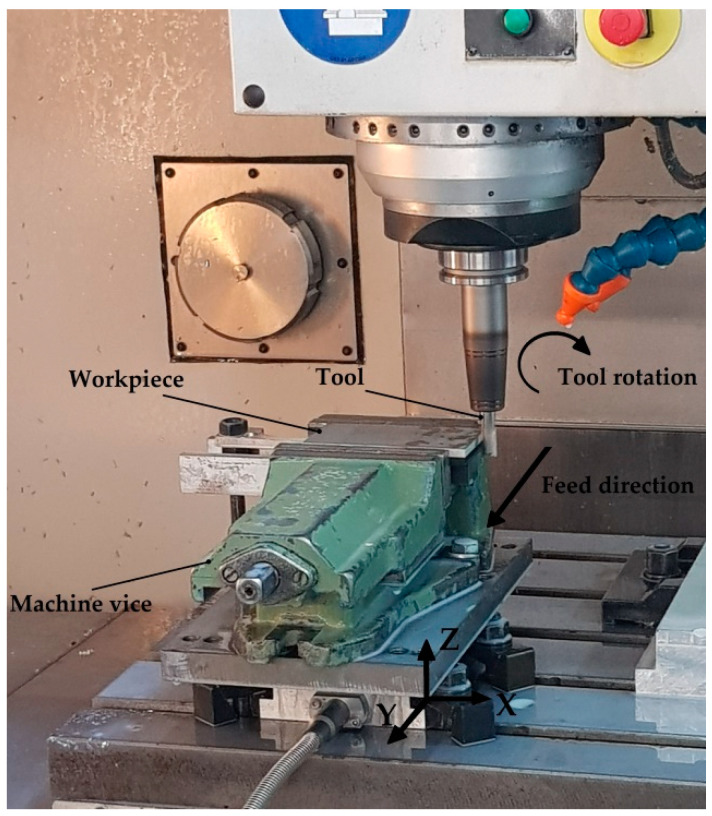
Scheme of cutting process.

Simultaneous circumferential milling using a VMC 800 HS machining centre (AVIA, Warsaw, Poland) was employed during the tests. The spindle performed right-hand rotation. The shorter edge of the sample (60 mm) was machined during the tests. Each sample was machined three times and the arithmetic mean of the three measurements was used in the analysis. Figure 2 presents the scheme of the milling process.

**Figure 3:**
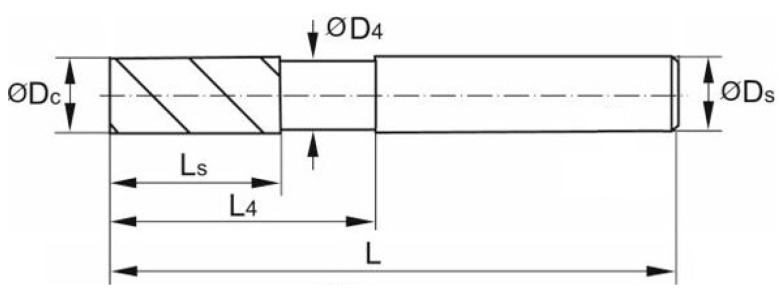
Geometry of endmill used [41].

Manufacturers of machining tools currently tend to make dedicated tools for machining specific types of materials. This makes it particularly difficult to determine the tools and process parameters suitable for machining both the facesheet and core materials. Careful selection of the tool, its geometry and the technological parameters of the process is an important part of the experiment. The experiment used a Garant double-edged, solid carbide endmill by Hoffman Group (Munich, Germany). The shape and dimensions of the tool are presented in Figure 3 and Table 2.

To examine the impact of the tool coating on the quality of the surface after milling, an identical endmill was used, this one coated with a 5-μm thick TiAlN coating, applied using the PVD method. The tools and coating type were selected due to their versatility (suitability for machining aluminum alloys and plastics). The machining was carried out using constant milling parameters: a milling speed v_c_ of 300 m/min, a feed rate f_z_ of 0.08 mm/blade, an axial milling depth a_p_ of 12 mm, and a radial milling depth a_e_ of 4 mm. The parameters were chosen based on values recommended by the manufacturer of the tool for machining aluminum alloys and polymers. The value of a_p_ was chosen so as to enable the simultaneous milling of both layers of the structure.

**Figure 4:**
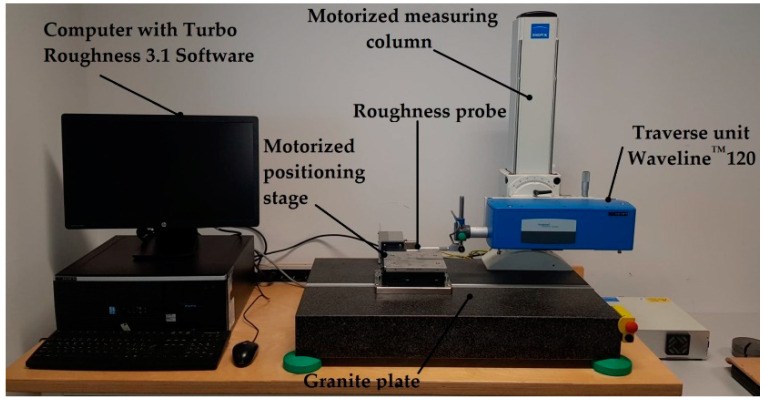
Scheme of experimental stand.

The roughness and topography of the surface were recorded using a 3D T8000 RC120-400 (Hommel-Etamic, Jena, Germany) surface roughness, waviness, and topography measurement device. The scheme of the experimental stand is shown in Figure 4.

Ra (arithmetic mean deviation of the assessed profile), Rz (maximum height of the profile), and Rmax (maximum roughness depth) surface roughness parameters [42], as well as the Sa (arithmetic mean height), Sz (maximum height), Sv (maximum pit height) and Sp (maximum peak height) surface topography parameters, were analyzed [43]. Measurements were taken at a distance equal to half of the thickness of the sample from the sample’s edge so that the measured section was positioned centrally to the layer being examined. The roughness and topography of the surface were measured separately for each layer in the structure, longitudinally to the feed direction. The test involved taking 320 measurements on the surface of each layer. The total length of the measurement l_t_, the length of the measurement section l_n_ and the length of the elementary section l_r_ were, respectively: l_t_ = 4.8 mm, l_n_ = 4.0 mm, l_r_ = 0.8 mm. The measuring interval was 15 µm. The measurement speed was 0.50 mm/s. The measurement time for one layer (320 measurement points) was about 2 h and 40 min.

**Figure 5:**
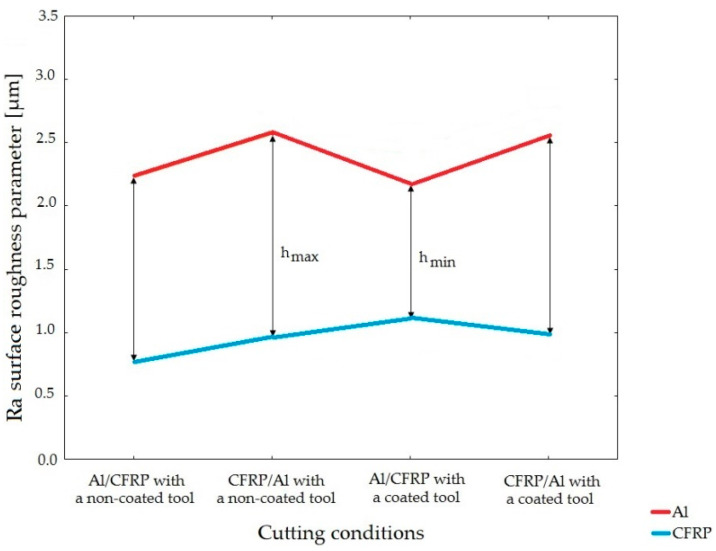
Determining the surface uniformity after milling based on selected surface roughness parameter.

The surface quality of the hybrid sandwich structures after milling may also be defined by the uniform or regularity of defect distribution on the machined surface. This is caused by the fact that the metal layer may have a different surface quality than the composite layer. Therefore, the surface uniformity was determined based on the roughness parameters of the surface, depending on the machining configuration and tool used (Figure 5).

It was assumed that the most uniform surface was represented by the lowest difference between the values of a given surface roughness parameter of each layer (h_min_), whereas the least uniform surface had the highest difference (h_max_).

## 3. Results

### 3.1. 2D Surface Roughness Parameters

**Figure 6:**
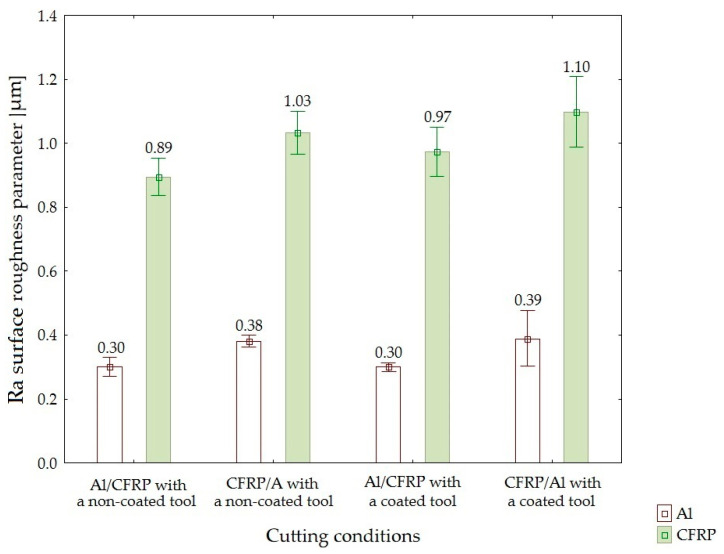
Values of Ra surface roughness parameter depending on the machining configuration and the type of tool.

**Figure 7:**
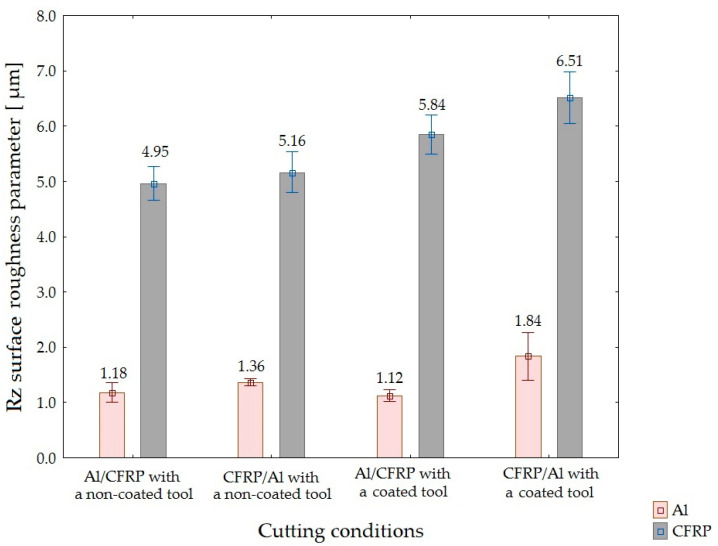
Values of Rz surface roughness parameter depending on the machining configuration and the type of tool.

**Figure 8:**
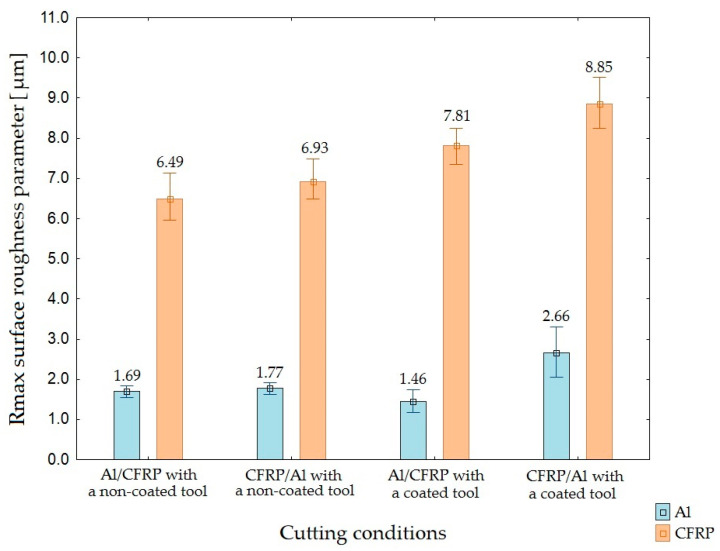
Values of Rmax surface roughness parameter depending on the machining configuration and the type of tool.

Figure 6, Figure 7 and Figure 8 illustrate the results of the roughness measurements of the 2D surface.

The minimum value of Ra parameter was obtained on the surface of the aluminum alloy, while the maximum value was observed on the surface of the CFRP composite. For all the cutting conditions, the values of Ra surface roughness parameter obtained on the surface of the CFRP composite were higher than the values of this parameter obtained for the aluminum alloy. The lowest values for the metal layer (0.30 µm) were measured in the following two machining configurations: Al/CFRP using a non-coated tool and Al/CFRP using a TiAlN-coated tool. The lowest value of this parameter for the composite layer was obtained when using the Al/CFRP machining configuration and a non-coated tool (0.89 µm). The highest values of Ra surface roughness parameter for the two materials (aluminum alloy—0.39 µm; CFRP composite—1.10 µm) were obtained in the CFRP/Al configuration, using a TiAlN-coated tool

### 3.2. Statistical Analysis of 2D Surface Roughness Parameters

To determine the impact of the machining configuration and the type of tool on the values of Ra, Rz, and Rmax surface roughness parameters, a two-factor analysis of variance (ANOVA) was carried out, the results of which are shown in Table 3, Table 4 and Table 5.

The data in Table 3 indicate that a statistically significant result for Ra surface roughness parameter measured at the surface of the aluminum alloy was recorded only for the machining configuration (F_1, 1276_ = 83.37; *p* < 0.01). Tool type and A × B interaction had similar values of the test statistic and had no significant impact on Ra surface roughness parameter. In the case of the Ra surface roughness parameter measured at the surface of the CFRP composite, statistically significant results were recorded for machining configuration (F_1, 1276_ = 190.52; *p* < 0.01) and tool type (F_1, 1276_ = 59.51; *p* < 0.01). The impact of the interaction between the two factors was statistically insignificant in this case as well.

Table 4 lists the results of ANOVA for the Rz surface roughness parameter. The analysis of these results indicates that the machining configuration, type of tool used, and the interaction between these variables had a statistically significant impact on the highest value of the roughness profile of the aluminum alloy and CFRP composite. In the case of the aluminum alloy, the machining configuration had the most significant impact on Rz surface roughness parameter (F_1, 1276_ = 66.42; *p* < 0.01), whereas the tool type had the least significant impact (F_1, 1276_ = 14.32; *p* < 0.01). ANOVA indicated that the tool type had the most significant impact on Rz surface roughness parameter for the CFRP composite (F_1, 1276_ = 404.2274; *p* < 0.01), while the A × B interaction had the least impact (F_1, 1276_ = 18.12; *p* < 0.01).

### 3.3. Surface Topography

**Figure 9:**
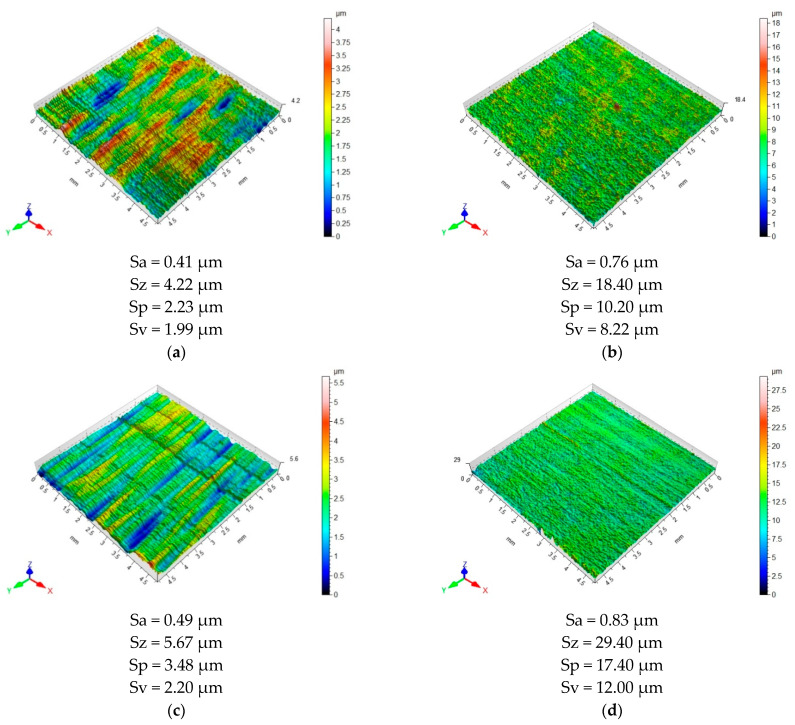
Surface topography after milling: (**a**) aluminum alloy in Al/CFRP configuration using a non-coated tool, (**b**) CFRP composite in Al/CFRP configuration using a non-coated tool, (**c**) aluminum alloy in CFRP/Al configuration using a non-coated tool, and (**d**) CFRP composite in CFRP/Al composite configuration using a non-coated tool.

**Figure 10:**
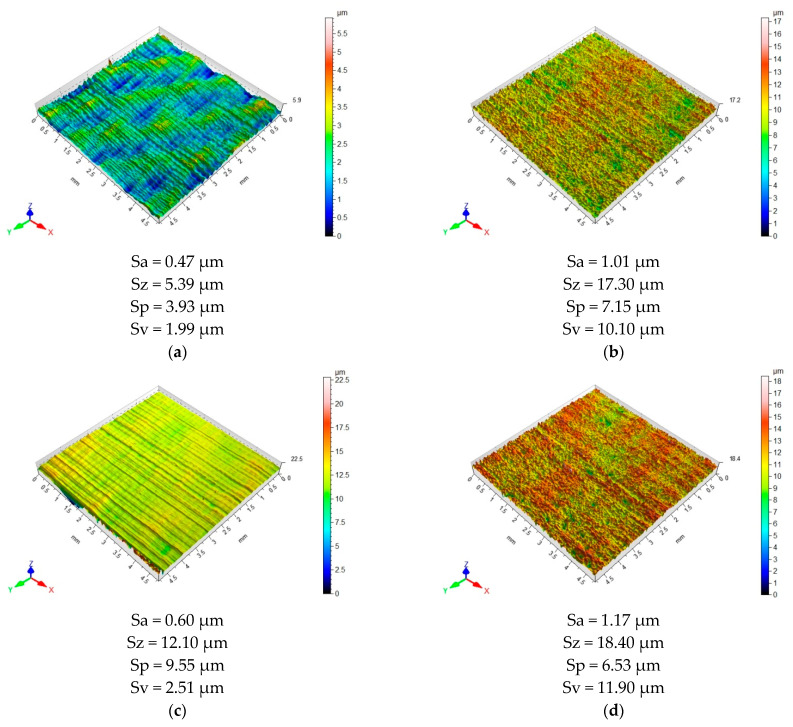
Surface topography after milling: (**a**) aluminum alloy in Al/CFRP configuration using a coated tool, (**b**) CFRP composite in Al/CFRP configuration using a coated tool, (**c**) aluminum alloy in CFRP/Al configuration using a coated tool, and (**d**) CFRP composite in CFRP/Al composite configuration using a coated tool.

Figure 9 and Figure 10 show 3D maps illustrating the surface topography of the samples after machining. The surface topography of each layer making up the sandwich structure is shown, depending on the machining configuration and tool used.

The surface topography of the metal layer of a sample milled in the Al/CFRP configuration using a non-coated tool (Figure 9a) was characterized by a directed arrangement of irregularities. Projections and indentations were both regular and random. Numerous grooves and ridges were also visible, diversifying the roughness of the surface. In the case of the CFRP composite (Figure 9b), a unidirectional surface with a random arrangement of irregularities was obtained. The values of the Sa, Sz, Sp, and Sv parameters at the surface of the aluminum alloy were 46%, 71%, 78%, and 76% lower, respectively, than at the surface of the composite layer.

After machining the aluminum alloy in the CFRP/Al configuration with a non-coated tool, a directed surface with a mixed structure was produced (Figure 9c). The surface topography was characterized by a diverse arrangement of irregularities. The surface of the CFRP composite was unidirectional, with a random, regular distribution of irregularities (Figure 9d). The values of Sa, Sz, Sp, and Sv parameters measured at the surface of the metal layer were nearly 41, 81%, 80%, and 82% lower, respectively, than the parameters measured at the surface of the composite layer.

Milling using the Al/CFRP configuration and a TiAlN-coated tool produced a directed structure with a determined distribution of irregularities on the surface of the aluminum alloy (Figure 10a). The irregularities were evenly distributed and spaced. In the case of CFRP composite (Figure 10b), a unidirectional surface with a random arrangement of irregularities was obtained. The microgeometry of the surface demonstrated a minor difference in the elevation of the elements of topography. The values of Sa, Sz, Sp, and Sv parameters measured at the surface of the aluminum alloy were 53%, 66%, 45%, and 80% lower than those for the composite layer, respectively.

Milling the metal layer with the CFRP/Al configuration and a coated tool produced a unidirectional topography with a regular arrangement of irregularities. The surface featured regular deformations: straight-line tracks spaced at similar intervals (Figure 10c). The topography of the composite surface was characterized by a unidirectional arrangement of irregularities. Numerous randomly arranged projections and indentations were visible (Figure 10d). In this case, the Sa and Sz values were 95% and 52% higher than in the case of the surface of the aluminum alloy, respectively. As regards the Sp value for the composite layer, the peaks of micro-irregularities were flattened: The Sp parameter was 32% lower, from 9.55 µm to 6.53 µm. The deepest indentation of the 3D profile (Sv) increased nearly fourfold.

### 3.4. Surface Uniformity

The surface uniformity of the two-layer structures after machining was determined based on the values of Ra, Rz, and Rmax surface roughness parameters.

**Figure 11:**
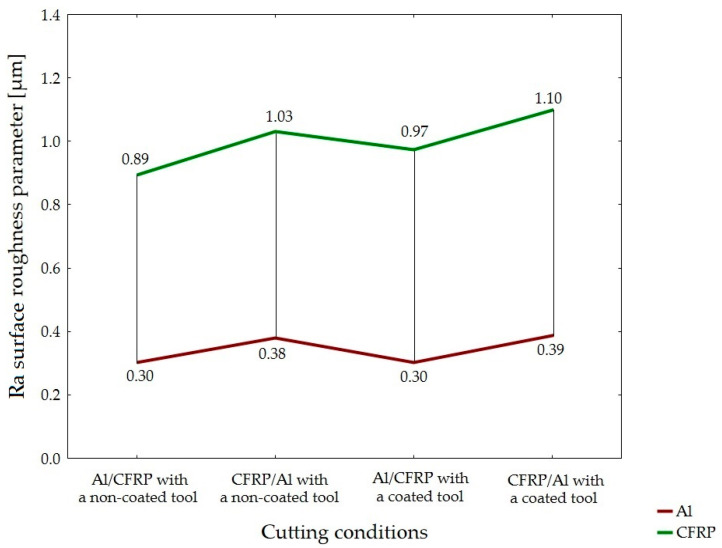
Surface uniformity based on the value of Ra surface roughness parameter obtained depending on the machining configuration and the type of tool.

In the case of Ra surface roughness parameter, the lowest difference between the layers was observed when using the Al/CFRP configuration and a non-coated endmill, whereas the highest difference was observed when using the CFRP/Al configuration and a TiAlN-coated tool (Figure 11).

**Figure 12:**
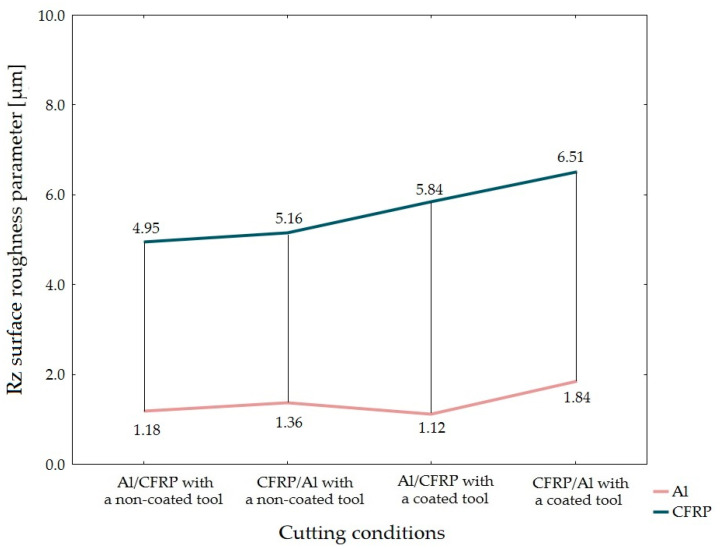
Surface uniformity based on the value of Rz surface roughness parameter obtained depending on the machining configuration and the type of tool.

**Figure 13:**
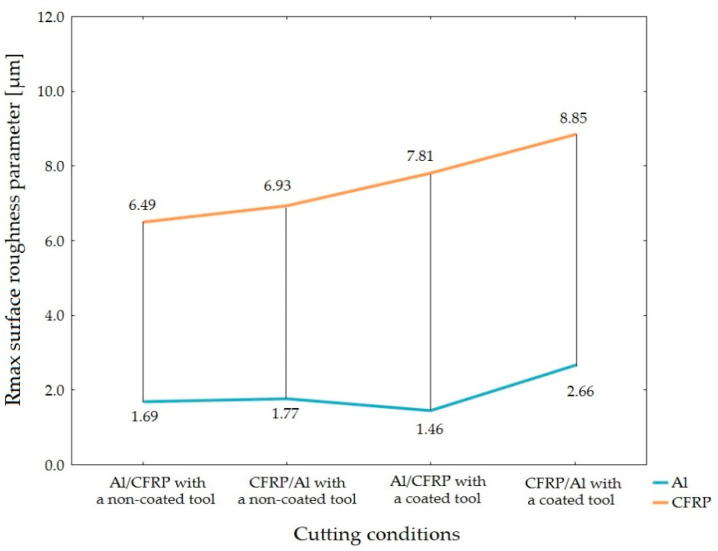
Surface uniformity based on the value of Rmax surface roughness parameter obtained depending on the machining configuration and the type of tool.

Based on the values of Rz and Rmax surface roughness parameter, it can be ascertain that the Al/CFRP configuration with a non-coated tool produced the highest surface uniformity (lowest differences between the values obtained for both layers of the sandwich structure), whereas the Al/CFRP configuration plus a coated tool were characterized by the lowest surface uniformity (Figure 12 and Figure 13).

Currently, no standards or laws exist that would regulate the acceptable surface quality of sandwich structures after machining. Many researchers have attempted to forecast the roughness of the fiber composite surfaces by creating theoretical models and comparing them to the results of experiments [44,45]. However, there are no standards or papers dealing with hybrid layered structures. Based on the authors’ experience in studying the surface quality of sandwich structures after machining, a coefficient that specifies the level of uniform of surface roughness after machining such materials was created. The coefficient was designated I_R_ and is calculated as follows:

(1) $$I_{R}=\frac{\left|R_{\mathrm{Al}}-R_{\mathrm{CFRP}}\right|}{\left|R_{\mathrm{Al}}+R_{\mathrm{CFRP}}\right|}$$

where

I_R_ is the surface uniformity coefficient;
R_Al_ is the aluminum alloy surface roughness parameter;
R_CFRP_ is the CFRP composite surface roughness parameter.

The difference between the values of selected surface roughness parameters of the layers’ surfaces was referenced against the total surface roughness of the sandwich structure. This enables the coefficient I_R_ to assess the surface uniformity of sandwich structure. By using any value of the given surface roughness parameter in formula (1), it can be observed that a higher difference between the values obtained for the aluminum alloy and the CFRP composite results in a lower uniform of the surface of the sandwich structure (h_max_) and a higher value of I_R_. Therefore, for the purposes of predicting the surface uniformity of sandwich structures after machining, it should be assumed that the lower the value of I_R_ (I_R_ → min), the higher the surface uniformity of the sandwich structure (h_min_).

Table 6 shows the calculated values of the coefficient I_R_ for the analyzed surface roughness parameters obtained for the adopted cutting conditions.

Analyzing the results in Table 6, it can be seen that the highest I_R_ values for the considered surface roughness parameters were obtained after milling in the Al/CFRP configuration using a coated tool. The lowest surface uniformity obtained for Rz and Rmax parameters was obtained for the same machining configuration and tool used. However, the minimum I_R_ values and the highest surface uniformity were not obtained for the same cutting conditions.

## 4. Discussion

The objective of this study was to determine the impact of machining configuration and tool type on the surface quality of the sandwich structure after milling. The results of the study indicate that similar values were obtained for the Rz and Rmax surface roughness parameters. The minimum and maximum values of the Ra surface roughness parameter were not obtained for the same cutting conditions, as was the case for the other parameters. This may have resulted from the fact that the Ra surface roughness parameter does not account for the presence of defects typical of fiber-reinforced polymers [46].

Using the CFRP/Al machining configuration and a coated tool resulted in a higher value of Ra surface roughness parameter on the aluminum alloy and the CFRP surfaces. However, the statistical analysis indicated that the machining configuration was the only variable that had a statistically significant impact on the value of Ra surface roughness parameter obtained for the metal layer. The statistically significant variables for the composite layer were the machining configuration and the tool type. The interaction of these factors was statistically insignificant for both materials.

The results indicate that a composite material has a lower machinability—for all considered cutting conditions, lower values of the tested parameters were obtained for the surface of the metal layer. This was due to the heterogeneity and abrasive properties of the composite material and the presence of typical defects on its surface (including fiber pull-out and matrix cracking) [47]. The values of Rz and Rmax surface roughness parameters were higher for the CFRP/Al configuration than the Al/CFRP configuration, for both the aluminum alloy and the CFRP composite [48]. Obtaining higher values of the surface roughness parameters in the CFRP/Al configuration is due to the different properties of the materials forming the sandwich structure. The aluminum layer above the composite material stiffened the workpiece, making the machining more stable. Using a coated tool reduced the surface roughness of the aluminum alloy in the Al/CFRP configuration, while it increased the value in the CFRP/Al configuration. Using a coated tool with the composite layer resulted in higher values of Rz and Rmax surface roughness parameters in both configurations. This was the result of the thicker endmill material and the rounding of the milling edge caused by the tool coating [49,50]. The machining configuration had the most significant impact on the values of Rz and Rmax surface roughness parameter for the aluminum alloy, whereas the tool type was the major factor in the case of the CFRP composite. Different values of the tested surface roughness parameters for aluminum alloy and CFRP composite was also due to the anisotropy of the sandwich structure. The tool encountered different cutting resistances during machining—when cutting the CFRP composite, which has a lower density compared to aluminum alloy, a sudden change in cutting resistance occurred, and the tool was pulled deep into the workpiece. The result was the occurrence of a non-uniform quality on the surface of the sandwich structure.

The analysis of surface topography indicated that in most cases higher 3D surface roughness parameters were obtained for the composite layer. In addition, the topographies of the aluminum alloy and CFRP composite surfaces had different micro-irregularity arrangements.

The study went on to create a coefficient for assessing the surface uniformity of sandwich structures. Based on the values of the surface roughness parameters obtained through experimentation, differences in the quality of the layers that made up the sandwich structure were calculated for each variant. The results were compared against the calculated values of the newly-created coefficient I_R_, leading to the assumption that the minimal value of I_R_ determines the most uniform quality of a sandwich structure. Analysis of the results showed that the cutting conditions (machining configuration and type of tool) allowing the lowest values of surface roughness parameters for the materials forming the structure did not guarantee the highest surface uniformity. This shows that surface uniformity is not the same as surface roughness and should still be considered separately. Therefore, it is necessary to continue research aimed at finding a tool and machining conditions to achieve surface roughness and surface uniformity at similar acceptable levels.

## 5. Conclusions

The following conclusions have been formulated based on the tests and analysis:

1. The composite layer had poorer surface quality than the aluminum layer.
2. The Ra surface roughness parameter is the least suitable of all analyzed surface roughness parameters for assessing the surface quality of sandwich structures after machining.
3. The CFRP/Al configuration increased the values of Ra, Rz and Rmax surface roughness parameters on the surfaces of both materials.
4. The tool coating did not affect the values of Ra parameter obtained on the surface of the aluminum alloy. For the CFRP composite, the presence of the TiAlN coating led to higher values of this parameter.
5. In most cases, a coated tool increased the Rz and Rmax surface roughness parameters.
6. The CFRP/Al configuration and a coated tool increased the values of Sa, Sz, Sp, and Sv 3D surface roughness parameters in the majority of cases.
7. Using the Al/CFRP configuration and a non-coated tool are recommended to receive the most uniform surface of the sandwich structure.
8. Milling with the Al/CFRP configuration and a TiAlN-coated tool resulted in the lowest surface uniformity.

## Figures and Tables

**Table 1:** Selected properties of the CFRP composite used in the test [40].

Tensile strength R_m_	1900
Bending strength R_eg_ [MPa]	2050
Young’s modulus (E) [GPa]	135
Apparent interlaminar shear strength (ILSF) [MPa]	85

**Table 2:** Technical data of tool used [41].

No. of teeth	2
Through-coolant	No
Tool material	Solid carbon (90% WC, 10% Co)
Cutting edge ØD_c_	12 mm
Shank Ø D_s_.	12 mm
Recess Ø D_4_	11.8 mm
Flute length L_s_	26 mm
Overhang length L_4_ incl. recess	38 mm
Overall length L	83 mm
Helix angle λ_s_	45°
Rake angle γ	16°
Corner chamfer angle	45°
Corner chamfer width at 45°	0.10 mm

**Table 3:** Two-factor analysis of variance for Ra surface roughness parameter depending on the machining configuration and the type of tool.

Impact	Aluminum Alloy				
	SS	Df	MS	F	*p*-Value
A: Machining configuration	2.18	1	2.18	83.37	<0.01
B: Tool type	<0.01	1	<0.01	0.13	0.72
A × B interaction	<0.01	1	<0.01	0.12	0.73
Error	33.42	1276	0.03		
Total	35.61	1279			
**Impact**	**CFRP**				
	**SS**	**Df**	**MS**	**F**	***p*-Value**
A: Machining configuration	5.51	1	5.51	190.52	<0.01
B: Tool type	1.72	1	1.72	59.51	<0.01
A × B interaction	0.02	1	0.02	0.70	0.4
Error	36.89	1276	0.03		
Total	44.14	1279			

**Table 4:** Two-factor analysis of variance for Rz surface roughness parameter depending on the machining configuration and the type of tool.

Impact	Aluminum Alloy				
	SS	Df	MS	F	*p*-Value
A: Machining configuration	65.12	1	65.12	66.42	<0.01
B: Tool type	14.04	1	14.04	14.32	<0.01
A × B interaction	23.05	1	23.05	23.51	<0.01
Error	1250.55	1276	0.98		
Total	1352.76	1279			
**Impact**	**CFRP**				
	**SS**	**Df**	**MS**	**F**	***p*-Value**
A: Machining configuration	61.03	1	61.03	64.28	<0.01
B: Tool type	404.22	1	404.22	425.74	<0.01
A × B interaction	17.20	1	17.20	18.12	<0.01
Error	1211.51	1276	0.95		
Total	1693.96	1279			

**Table 5:** Two-factor analysis of variance for Rmax surface roughness parameter depending on the machining configuration and the type of tool.

Impact	Aluminum Alloy				
	SS	Df	MS	F	*p*-Value
A: Machining configuration	132.08	1	132.08	47.22	<0.01
B: Tool type	34.01	1	34.01	12.16	<0.01
A × B interaction	101.44	1	101.44	36.27	<0.01
Error	3569.08	1276	2.80		
Total	3836.61	1279			
**Impact**	**CFRP**				
	**SS**	**Df**	**MS**	**F**	***p*-Value**
A: Machining configuration	172.20	1	172.20	50.08	<0.01
B: Tool type	836.96	1	836.96	243.40	<0.01
A × B interaction	29.24	1	29.24	8.50	<0.01
Error	4387.60	1276	3.44		
Total	5426	1279			

**Table 6:** Values of the coefficient I_R_ for the considered surface roughness parameters depending on the machining configuration and the type of tool.

Cutting Conditions	Surface Roughness Parameter		
	Ra	Rz	Rmax
Al/CFRP with a non-coated tool	0.50	0.61	0.58
CFRP/Al with a non-coated tool	0.46	0.53	0.59
Al/CFRP with a coated tool	0.53	0.68	0.69
CFRP/Al with a coated tool	0.48	0.58	0.54

## Data Availability

The raw/processed data required to reproduce these findings cannot be shared at this time due to technical or time limitations. Data can be made available on individual request.
